# Partial 18S ribosomal DNA sequences of nematode species collected in South Korea

**DOI:** 10.17912/micropub.biology.000556

**Published:** 2022-04-19

**Authors:** Jihye Yoon, Daye Kwon, Jun Kim, Junho Lee

**Affiliations:** 1 . Institute of Molecular Biology and Genetics, Seoul National University, Seoul 08826, Korea; 2 . Department of Biological Sciences, Seoul National University, Seoul 08826, Korea; 3 . Research Institute of Basic Sciences, Seoul National University, Seoul 08826, Korea; 4 IMBG, RIBS, Department of Biological Sciences, Seoul National University, Seoul 08826, Korea

## Abstract

Free-living nematodes are important model organisms in biology, and they can be collected from various materials including rotten fruit, plant, and soil. In order to explore the diversity of free-living nematodes in South Korea, we collected and isolated nematodes from rotten fruit matter from Seoul and Jeju island. Here, we report partial 18S ribosomal DNA sequences of the nematodes that we collected in South Korea between June and July of 2021. Three newly identified sequences are included.

**Figure 1. Pairwise alignments between rDNA sequences of the three previously reported species and the new nematode sequences identified in this study.**
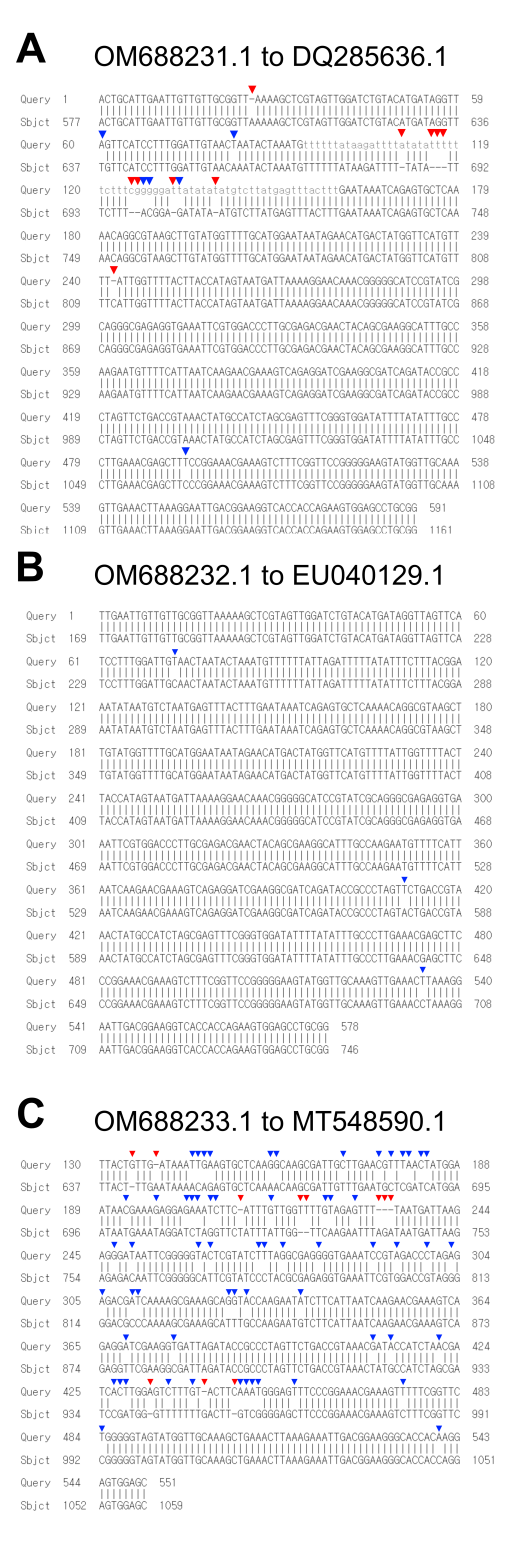
Red arrowheads represent gaps and blue arrowheads represent mismatches. (A) Alignment of our OM688231.1 sequence to rDNA sequence of *Panagrolaimus cf. rigidus* (DQ285636.1). (B) Alignment of our OM688232.1 sequence to rDNA sequence of *Panagrolaimus* sp. FL-SType-9 (EU040129.1). (C) Alignment of our OM688233.1 sequence to rDNA sequence of *Oscheius chongmingensis* (MT548590.1).

## Description

Free-living nematodes are important model organisms in biology, and they can be collected from various materials including rotten fruit, plant, and soil (Barrière and Félix 2005; Nigon and Félix 2017). In order to explore the diversity of free-living nematodes in South Korea, we collected and isolated nematodes from rotten fruit matter from Seoul and Jeju island. Then, we cultured them and extracted DNA to identify species through partial ribosomal RNA gene (rDNA) sequencing. Here, we report a subset of species that we collected in South Korea during June and July of 2021 ( **Table 1** ).

Among 30 different samples collected from 14 rotten fruits, *Caenorhabditis briggsae* was the major species (n=15), followed by *Chylorhabditis epuraeae* (n=5). While most of the fruits had been occupied by one dominant species, we observed multiple species from 2 out of the 14 fruits (Samples 1 and 9 in **Table 1** ). In addition, the majority (n=24) of the samples matched with the preexisting species and their rDNA sequences on the NCBI database, including *Auanema freiburgensis* ( KY680647.1 ) (Isolate 1), *Caenorhabditis briggsae* ( MN519141.1 ) (Isolates 2–16) *, Chylorhabditis epuraeae* ( LC570777.1 ) (Isolates 17–21) *, Oscheius sp. IR* ( MW667590.1 ) (Isolate 24) *,* and *Oscheius tipulae* ( MH983026.1 ) (Isolates 25–26).

Interestingly, we identified three species (n=6) that do not have perfect matching preexisting rDNA sequences on the NCBI database (Figure 1). Initially, these samples were determined to be closest to *Panagrolaimus cf. rigidus* ( DQ285636.1 ; n=2; Isolates 27–28), *Panagrolaimus sp.* FL-SType-9 ( EU040129.1 ; n=2; Isolates 29–30) and *Oscheius chongmingensis* ( MT548590.1 ; n=2; Isolates 22–23). However, each of their identity matched with *Panagrolamus cf. rigidus* by 98% (579/592; Figure 1A) *,* *Panagrolaimus* sp. FL-SType-9 by 99% (575/578; Figure 1B), and *Oscheius chongmingensis* by 83% (355/428; Figure 1C). The two isolates obtained from each species had identical variants and such variants were confirmed manually by examining graphical variant peaks. Thus, we concluded that the mismatch in identity in each species could be seen as real variants and not a sequencing error. This suggests that they are either novel species, species without reported rDNA sequences, or genetically divergent individuals of previously reported species. Therefore, in this paper, we report three rDNA sequences that have not been reported earlier. Their partial rDNA sequences are available in the NCBI database under accession numbers OM688231–OM688233.

**Table 1. Sampling information**

**Table:** 

**Isolate**	**Sample**	**Date**	**(Latitude,** **longitude)**	**Material**	**Location**	**Closest species name**	**Sequence ID**	**Accession of newly identified sequences**
1	1	7/9/2021	(33.26189,126.42172)	Tangerine	Jeju island	*Auanema freiburgensis*	KY680647.1	
2	2	6/29/2021	(37.45424,126.95374)	Persimmon	Seoul	*Caenorhabditis briggsae*	MN519141.1	
3	2	6/29/2021	(37.45424,126.95374)	Persimmon	Seoul	*Caenorhabditis briggsae*	MN519141.1	
4	2	6/29/2021	(37.45424,126.95374)	Persimmon	Seoul	*Caenorhabditis briggsae*	MN519141.1	
5	2	6/29/2021	(37.45424,126.95374)	Persimmon	Seoul	*Caenorhabditis briggsae*	MN519141.1	
6	3	6/29/2021	(37.45424,126.95374)	Persimmon	Seoul	*Caenorhabditis briggsae*	MN519141.1	
7	3	6/29/2021	(37.45424,126.95374)	Persimmon	Seoul	*Caenorhabditis briggsae*	MN519141.1	
8	3	6/29/2021	(37.45424,126.95374)	Persimmon	Seoul	*Caenorhabditis briggsae*	MN519141.1	
9	4	7/2/2021	(37.56210,126.89571)	Armenian plum	Seoul	*Caenorhabditis briggsae*	MN519141.1	
10	4	7/2/2021	(37.56210,126.89571)	Armenian plum	Seoul	*Caenorhabditis briggsae*	MN519141.1	
11	4	7/2/2021	(37.56210,126.89571)	Armenian plum	Seoul	*Caenorhabditis briggsae*	MN519141.1	
12	1	7/9/2021	(33.26189,126.42172)	Tangerine	Jeju island	*Caenorhabditis briggsae*	MN519141.1	
13	5	7/9/2021	(33.26191,126.42175)	Tangerine	Jeju island	*Caenorhabditis briggsae*	MN519141.1	
14	5	7/9/2021	(33.26191,126.42175)	Tangerine	Jeju island	*Caenorhabditis briggsae*	MN519141.1	
15	5	7/9/2021	(33.26191,126.42175)	Tangerine	Jeju island	*Caenorhabditis briggsae*	MN519141.1	
16	6	7/9/2021	(33.26183,126.42200)	Tangerine	Jeju island	*Caenorhabditis briggsae*	MN519141.1	
17	7	7/23/2021	(37.55013,127.16773)	Chinese plum	Seoul	*Chylorhabditis epuraeae*	LC570777.1	
18	7	7/23/2021	(37.55013,127.16773)	Chinese plum	Seoul	*Chylorhabditis epuraeae*	LC570777.1	
19	7	7/23/2021	(37.55013,127.16773)	Chinese plum	Seoul	*Chylorhabditis epuraeae*	LC570777.1	
20	8	7/19/2021	(37.56202,126.89667)	Persimmon	Seoul	*Chylorhabditis epuraeae*	LC570777.1	
21	8	7/19/2021	(37.56202,126.89667)	Persimmon	Seoul	*Chylorhabditis epuraeae*	LC570777.1	
22	1	7/9/2021	(33.26189,126.42172)	Tangerine	Jeju island	*Oscheius chongmingensis*	MT548590.1	OM688233
23	9	7/9/2021	(33.26192,126.42178)	Tangerine	Jeju island	*Oscheius chongmingensis*	MT548590.1	OM688233
24	10	7/9/2021	(33.26193,126.42194)	Tangerine	Jeju island	*Oscheius* sp. IR	MW667590.1	
25	9	7/9/2021	(33.26192,126.42178)	Tangerine	Jeju island	*Oscheius tipulae*	MH983026.1	
26	11	7/9/2021	(33.26187,126.42205)	Tangerine	Jeju island	*Oscheius tipulae*	MH983026.1	
27	12	6/9/2021	(33.26186,126.42227)	Tangerine	Jeju island	*Panagrolaimus cf. rigidus*	DQ285636.1	OM688231
28	13	6/9/2021	(33.26143,126.42175)	Tangerine	Jeju island	*Panagrolaimus cf. rigidus*	DQ285636.1	OM688231
29	14	6/9/2021	(33.26197,126.42221)	Tangerine	Jeju island	*Panagrolaimus* sp. FL-SType-9	EU040129.1	OM688232
30	14	6/9/2021	(33.26197,126.42221)	Tangerine	Jeju island	*Panagrolaimus* sp. FL-SType-9	EU040129.1	OM688232

## Methods

For the nematode collection, we first collected rotten fruit matter - tangerine, persimmon, Armenian plum, Chinese plum - from Seoul and Jeju island. We isolated the nematodes on the day of collection. The fruits were washed and mixed with distilled water. After centrifugation, about 90% of the supernatant was removed and the pellet was mixed with the remaining supernatant. Then, the solution containing nematodes from each fruit was pipetted onto an NGM plate, and the nematodes were isolated to a new plate after 10 to15 minutes for further growth. Once initial nematodes laid eggs, we singled-out 3 adult female or hermaphrodite individuals from each plate onto new NGM plates.

After the populated growth of singled-out samples, we performed partial rDNA sequencing with nem1 and nem2 as primers (Foucher and Wilson 2002). Up to 20 bp at the beginning and the end of the sequence were trimmed by hand to remove ambiguities and primer sequences. The sequence was also trimmed for low quality. Finally, for the species diagnosis, we searched for matching sequences and species using NCBI BLAST (NCBI Resource Coordinators 2018).

## Reagents

Primer set (5' to 3')nem1 (forward): GCA AGT CTG GTG CCA GCA GCnem2 (reverse): CCG TGT TGA GTC AAA TTA AG
